# Circadian Rhythm Disorders Aggravate Periodontitis by Modulating BMAL1

**DOI:** 10.3390/ijms24010374

**Published:** 2022-12-26

**Authors:** Xiaomeng Liu, Niuben Cao, Xinchan Liu, Yu Deng, Yu Xin, Ruobing Fu, Xirui Xin, Yubo Hou, Weixian Yu

**Affiliations:** 1 Department of Periodontics, School of Dentistry, Jilin University, Changchun 130021, China; 2 Department of Oral Implantology, School of Dentistry, Jilin University, Changchun 130021, China; 3 Jilin Provincial Key Laboratory of Tooth Development and Bone Remodeling, Changchun 130021, China

**Keywords:** circadian rhythm disturbances, circadian clock, BMAL1, periodontitis

## Abstract

Circadian rhythms regulate the body’s homeostasis through the temporal control of tissue-specific circadian rhythm control genes. Circadian rhythm disorders (CRD) affect the expression levels of circadian rhythms-associated genes in brain and muscle aryl hydrocarbon receptor nuclear translocator-like-1(BMAL1), which is thought to contribute to metabolic disorders and an altered immune system. However, the relationship between CRD and the development of periodontitis was poorly reported. Therefore, this study aimed to investigate the role played by BMAL1 in periodontitis. We used a modified multi-platform approach (MMPM) to induce circadian rhythm disturbances in rats to investigate the role of BMAL1 in periodontitis. Our results showed significant downregulation of BMAL1 in the CRD with periodontitis group, significant resorption of alveolar bone, increased osteoclast differentiation, and upregulation of the inflammatory signaling molecule NF-κB. In addition, apoptosis and oxidative stress levels were increased in periodontal tissues. Collectively, our study suggests that BMAL1 is a key regulator in periodontitis exacerbated by CRD and that CRD may lead to the downregulation of BMAL1, thereby exacerbating oxidative stress and apoptosis in periodontal tissues. Our study found that BMAL1 may be associated with the progression of periodontitis and provides a new perspective on the treatment of periodontitis.

## 1. Introduction

The circadian clock, present in all cells and organs of mammals, is an internal timing system that plays an important function in altering regular physiological activity [1,2]. Circadian rhythms are inherent survival instincts of organisms that enable them to predict and prepare for predictable environmental changes caused by the daily rotation of the Earth [3,4]. Circadian rhythm disorders have become a common phenomenon in modern society, and their occurrence is often associated with shift work, sleep disorders and various inflammatory diseases [5,6]. They can aggravate patients’ diseases and induce the production of inflammatory cells and inflammatory factors. [7]. Circadian rhythm disorders are associated with a variety of diseases and increase the risk of many diseases, such as obesity, cancer, and cardiovascular and metabolic diseases [8,9]. CRD may disrupt the immune defense system of the organism and promote the development of inflammatory responses [6]. According to epidemiological studies, CRD resulted in harmful effects on health by suppressing immunity and promoting inflammation [10,11]. The circadian clocks are composed of interacting genes in a network of oscillating transcripts widely distributed in mammalian cells [12]. Notably, the rhythmic genes contained in the circadian clock participate in modulating the gene expression related to cell physiology and metabolism [13,14].

Major regulators of mammalian circadian rhythmicity are thought to include the transcription-translation feedback loop (TTFL), which drives the periodic expression of clock gene products [15]. Positive regulation branch of the brain and muscle aryl hydrocarbon receptor nuclear translocator-like-1 (BMAL1) and Circadian Locomotor Output Cycles Kaput (CLOCK), and negative regulation branch of Periods (PER) and Cryptochromes (CRY) [14]. Post-translational modification of BMAL1 by acetylation, ubiquitination and phosphorylation plays a critical role in enhancing the molecular oscillation of biological clocks and regulating a variety of biological functions, including intercellular localization of biological clock-related molecules and precise temporal maintenance between the formation of the BMAL1:CLOCK complex and the repression of Per and Cry transcription [16]. The BMAL1 is an important part of the mammalian clock gene regulatory network and a sensitive point in the network. BMAL1 knockout has been shown to cause severe arrhythmias [17]. BMAL1 modulates other related rhythmic genes in vivo through a transcription-translation feedback loop and has an essential effect on molecular circadian oscillations [18,19]. BMAL1 is a unique core clock regulator and deletion of the BMAL1 gene not only causes circadian rhythm disorders but is also closely associated with the pathological processes of several diseases. Reduced BMAL1 expression has been reported to cause increased vascular uptake of low-density lipoprotein (LDL), induce endothelial cell dysfunction, and expression adhesion factors and pro-inflammatory mediators, leading to peripheral arterial disease [20].In addition, it was found that impaired BMAL1 expression and function in the liver can disrupt glucose metabolism homeostasis leading to chronic hepatic metabolic diseases [21]. More importantly, deletion of the BMAL1 gene can increase the incidence of diseases such as mandibular hypoplasia and cancer [22,23]. In contrast, activation of BMAL1 signaling molecules protects against cerebral ischemia-reperfusion injury in the hyperglycemic state [24]. This suggests that the activation of BMAL1 expression is essential for maintaining the healthy state of the organism. Notably, epidemiological studies have shown that CRD increases the risk and severity of periodontitis [11]. Although there is evidence for the involvement of BMAL1 in periodontitis, its molecular mechanisms in periodontitis are poorly understood. Therefore, this study aimed to investigate the role of BMAL1 in periodontitis.

## 2. Results 

### 2.1. CRD Model Was Constructed in Rats

**Figure 1:**
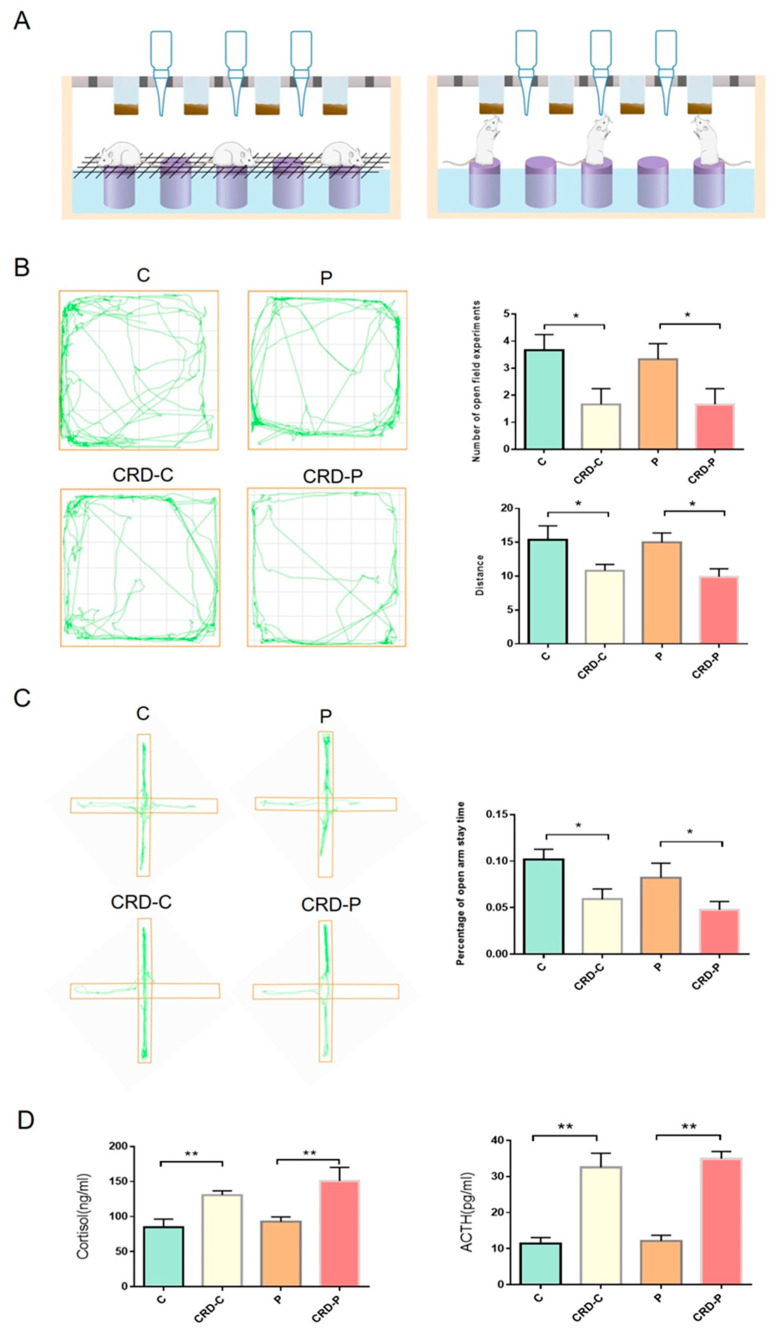
CRD model was constructed in rats. (**A**) CRD model diagram. (**B**) The results of the open field experiment. (**C**) Cross-elevation maze experiment. (**D**) Elisa’s results of cortisol and ACTH. CRD: circadian rhythm disorders; C: control; CRD-C: CRD without periodontitis. P: periodontitis group. CRD-P: CRD with periodontitis. Data are presented as the mean ± SD. *, *p* < 0.05; **, *p* < 0.01.

Firstly, we used a modified multiplatform approach to induce a CDR model in rats. (Figure 1A). The open-field experiment results showed that the rats in the CRD-C group entered the middle of the open field less often and walked shorter distances compared to the control group. Similarly, rats in the CRD-P group entered the center of the open space significantly less frequently and walked significantly shorter distances compared to the P group (Figure 1B). In addition, cross-elevation maze experiments showed that CRD-C and CRD-P groups had significantly shorter arms open than the C and P groups (Figure 1C). The results showed that CRD led to reduced behavioral capacity and improved stress response in rats. Our results showed that serum cortisol and ACTH levels were elevated in the CRD-C and CRD-P groups of rats compared to the C and P groups (Figure 1D). Consistently, CRD can activate the suprachiasmatic nucleus (SCN) in the hypothalamus, which, under the action of central rhythm controllers, leads to an increase in serum cortisol and ACTH levels through the hypothalamic–pituitary–adrenal axis (HPA) axis [25,26]. Our results showed that serum cortisol and ACTH levels were elevated in the CRD-C and CRD-P groups of rats than in the C and P groups (Figure 1D). The above results indicate that the animal model of CRD has been successfully constructed in this study.

### 2.2. CRD Promotes the Progression of Periodontitis

**Figure 2:**
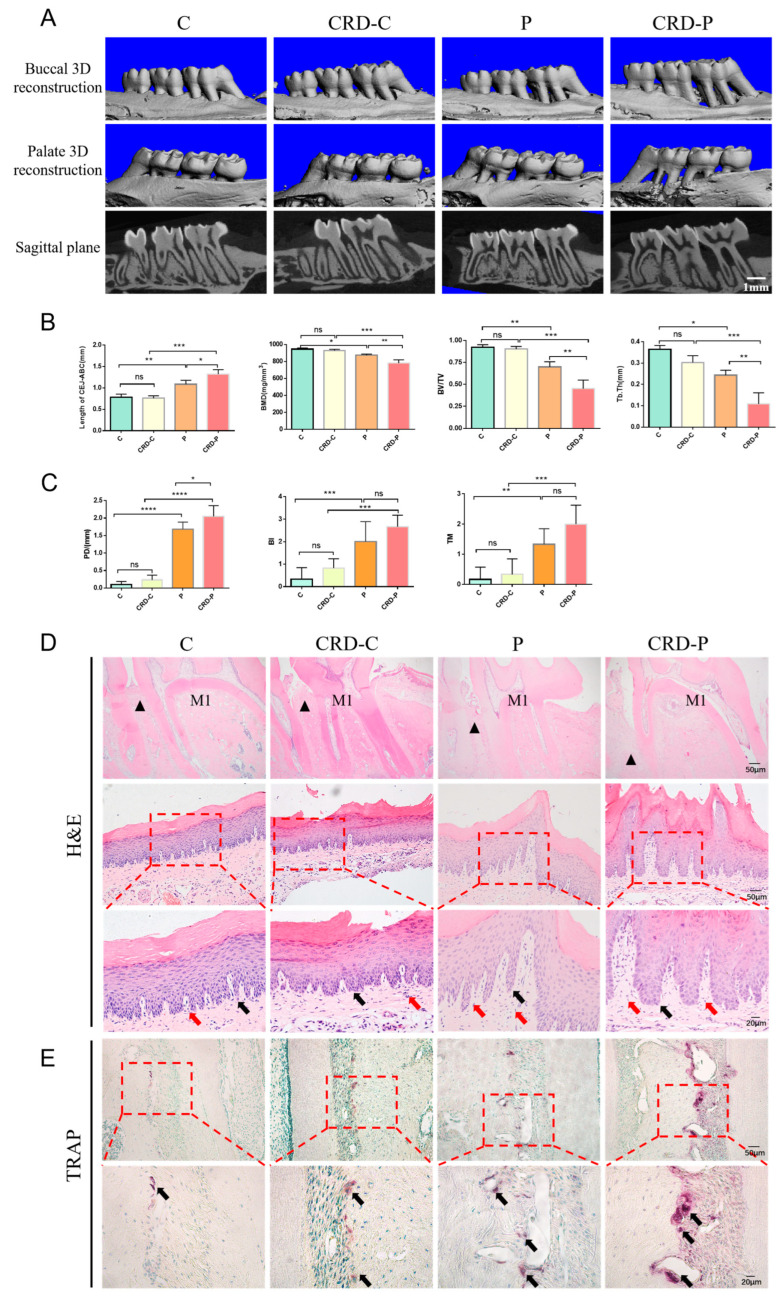
(**A**) Micro-CT (Scale bar = 1 mm). (**B**) BMD, BV/TV, Tb.Th, and the distance from CEJ to ABC were analyzed. (**C**) The results of the periodontal clinical index of rats in each group include pocket depth (PD), tooth mobility (TM), and bleeding index (BI). (**D**) H&E staining results of maxillary first molars (Scale bar = 200 μm). M1 indicates the maxillary first molar. The black triangle means the alveolar bone ridge and H&E staining results in gingival tissue of rat maxillary first molars at 200× and 400× magnification. The black arrows show inflammatory cells. The red arrows show the epithelial pegs. (**E**) Results of TRAP staining of maxillary first molars in rats at 200× and 400× magnification. The black arrows indicate osteoclasts. CRD: circadian rhythm disorders. C: control; CRD-C: CRD without periodontitis. P: periodontitis group. CRD-P: CRD with periodontitis. Data are presented as the mean ± SD. ns, not significant difference; * *p* < 0.05; ** *p* < 0.01; *** *p* < 0.001; **** *p* < 0.0001.

To study the effect of CRD on periodontitis, we constructed a periodontitis model by ligating the cervical part of the maxillary first molar in rats after successfully establishing a CRD model. Micro-CT results showed significant bone resorption in the alveolar crest and root bifurcation areas of maxillary first molars in rats with periodontitis compared to controls (Figure 2A,B). In addition, periodontal pocket probing depth, tooth mobility, and gingival bleeding index were significantly increased (Figure 2C), which indicated that the rat periodontitis model was successfully constructed in this study. Next, we continued to explore the influence of CRD factors on periodontitis progression. Our results showed that the distance from CEJ to ABC of the maxillary first molar increased in the CRD-P group rats than in the P group, while BMD, BV/TV, and Tb.Th decreased (Figure 2B), indicating that alveolar bone resorption was more severe in the CRD-P group rats. H&E staining showed that both the P and CRD-P groups exhibited epithelial peg elongation and extensive infiltration of inflammatory cells. Still, the attachment loss was more severe in the CRD-P group (Figure 2D). In addition, TRAP staining showed that a significantly higher number of activated osteoclasts were found on the alveolar bone surface of rats in the CRD-P group than in the P group (Figure 2E). This indicates that periodontitis progression may be promoted under the effect of CRD factors. Notably, there was no significant difference in the alveolar bone correlation coefficient and CEJ-to-ABC distance between the C and CRD-C groups. However, inflammatory cell infiltration in the gingival tissue of maxillary first molars was increased in the CRD-C group than in the C group, indicating that CRD may lead to altered levels of inflammation in periodontal tissue.

### 2.3. CRD Promotes the Progression of Periodontitis by Regulating BMAL1

**Figure 3:**
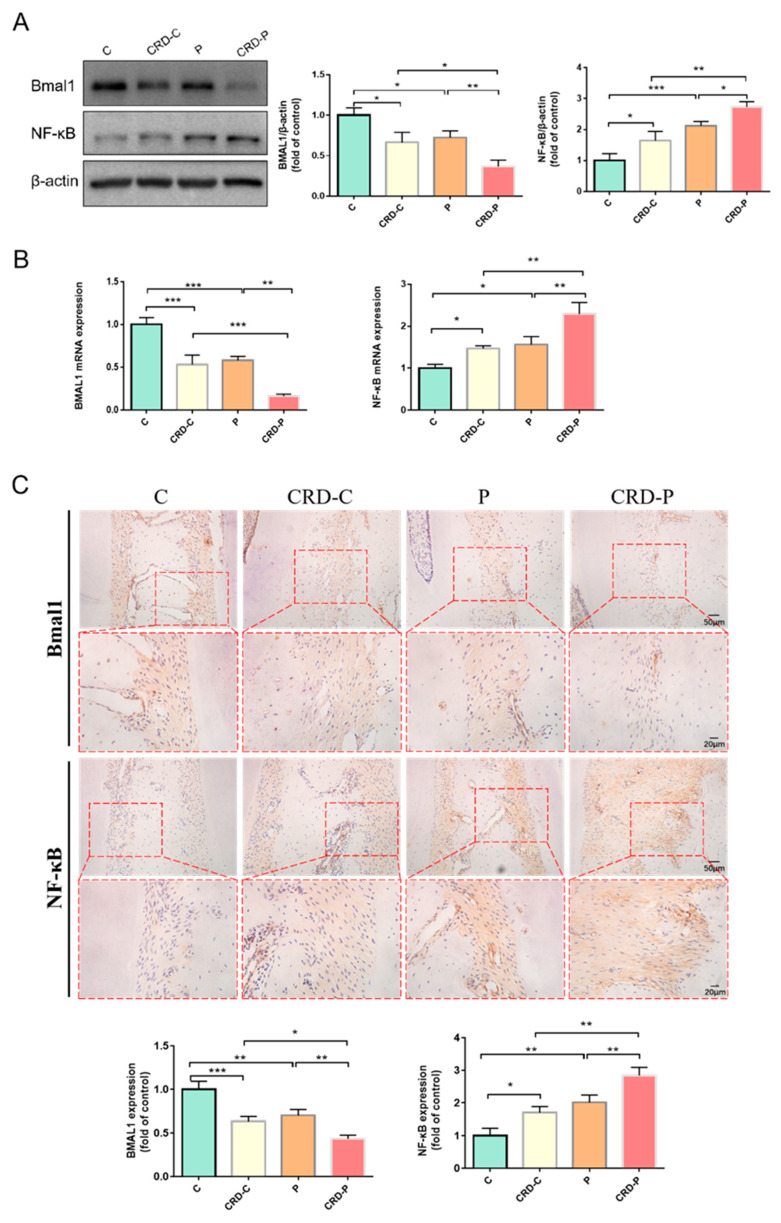
CRD promotes the progression of periodontitis by regulating BMAL1. (**A**) Western blot results and analysis of BMAL1 and NF-κB. (**B**) BMAL1 and NF-κB mRNA expression levels in gingival tissue of rat maxillary first molars. (**C**) Immunohistochemical staining of BMAL1, NF-κB in the periodontal tissue of rat maxillary first molars (Scale bar = 20 μm and Scale bar = 50 μm). CRD: circadian rhythm disorders. C: control; CRD-C: CRD without periodontitis. P: periodontitis. CRD-P: CRD with periodontitis. Data are presented as the mean ± SD. * *p* < 0.05; ** *p* < 0.01; *** *p* < 0.001.

Then we examined the mechanistic link between CRD and periodontitis, and previous literature reported that BMAL1 has a vital effect in maintaining circadian rhythm homeostasis and regulating a variety of cellular activities, while NF-κB signaling molecule is the foundation of the mutual modulation between the biological clock and inflammation. We speculated whether CRD exacerbates the occurrence and development of periodontitis through inflammatory signals. Our results showed that the expression level of BMAL1 was significantly lower in periodontal tissues of both CRD-C and P group rats than in the C group, suggesting that the reduced expression levels of BMAL1 are correlated with circadian rhythm disturbance and altered inflammation levels. More importantly, the expression level of BMAL1 in periodontal tissues was significantly lower in the CRD-P group compared with the P group, in comparison, the expression level of NF-κB was considerably higher (Figure 3A–C). The above results indicate that CRD may promote periodontitis progression via downregulating the expression of BMAL1.

### 2.4. CRD Exacerbates the Redox State of Periodontal Tissues

**Figure 4:**
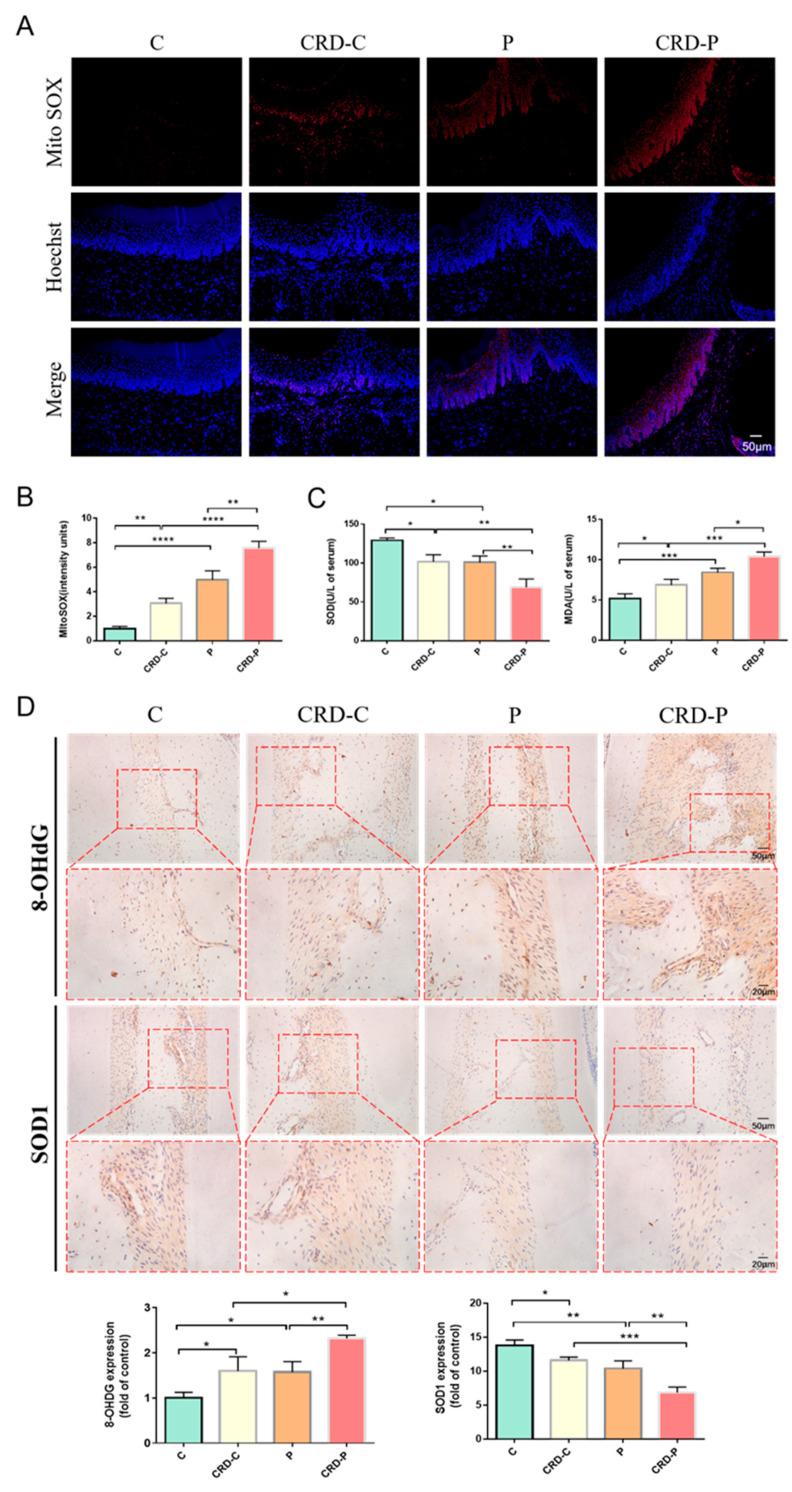
CRD exacerbates the redox state of periodontal tissues. (**A**,**B**) Mito SOX Red staining results and semiquantitative analysis (Scale bar = 50 μm). (**C**) The serum MDA levels and SOD activity of rats were examined. (**D**) Immunohistochemical staining of 8-OHdG and SOD1 in the periodontal tissue of rat maxillary first molars in each group (Scale bar = 20 μm and Scale bar = 50 μm). CRD: circadian rhythm disorders C: control; CRD-C: CRD without periodontitis. P: periodontitis group. CRD-P: CRD with periodontitis. Data are presented as the mean ± SD. * *p* < 0.05; ** *p* < 0.01; *** *p* < 0.001; **** *p* < 0.0001.

To further examine the molecular signaling mechanisms involved in CRD exacerbating periodontitis, we found that BMAL1 has been reported to have a crucial function in regulating intracellular redox status [27,28]. Given that OS may be a critical pathogenic factor in periodontitis, we next explored the role of BMAL1 in regulating the redox status of periodontal tissues. Superoxide dismutase 1 (SOD1) is an important antioxidant enzyme. 8-hydroxy-2′-deoxyguanosine (8-OHdG) is a biomarker of oxidative DNA damage. Our results showed that 8-OHdG expression levels were elevated in the periodontal tissues in the CRD-P group than in the P group, while the SOD1 expression levels were decreased. In addition, MDA is a recognized lipid peroxidation product for assessing oxidative stress and is the most investigated lipid peroxidation product in periodontitis. We found that serum MDA levels and ROS content in periodontal tissues were elevated in the CRD-P group rats than in the P group, while serum SOD activity was decreased. These data suggest that the CRD-P group exhibits more severe OS in the periodontal tissue (Figure 4A–D). Therefore, we recommend that BMAL1 downregulation and NF-κB hyperactivation may be one of the pathways through which CRD exacerbates oxidative damage in periodontal tissues.

### 2.5. Disturbed Circadian Rhythms Exacerbate Apoptosis in Periodontal Tissues

**Figure 5:**
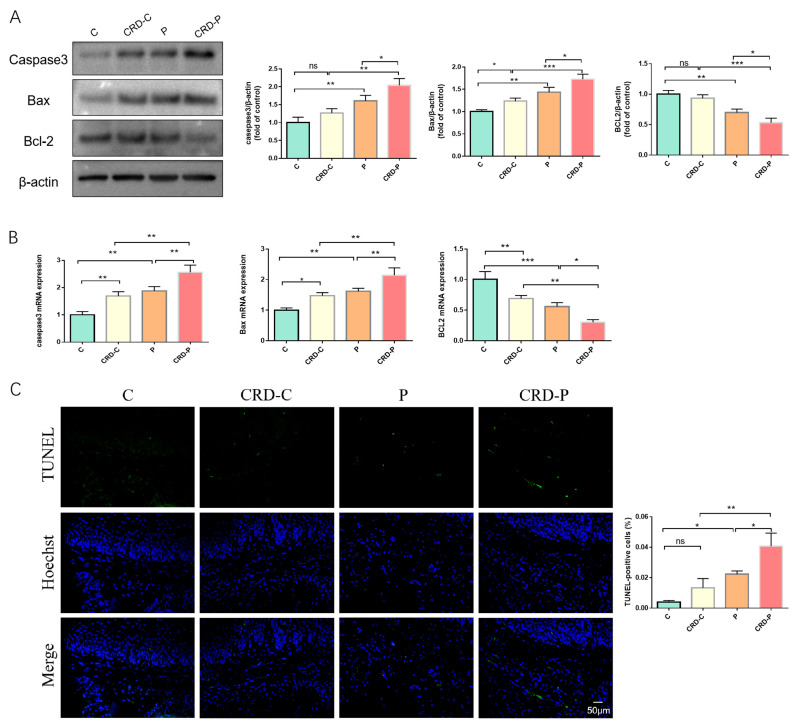
CRD exacerbates apoptosis in periodontal tissues. (**A**) Western blot results and analysis of Caspase3, Bax, and Bcl-2. (**B**) The apoptosis-related factor mRNA expression levels in the periodontal tissue of maxillary first molars. (**C**) TUNEL staining results. C: control. CRD-C: CRD without periodontitis. P: periodontitis group. CRD-P: CRD and periodontitis. Data are presented as the mean ± SD. ns, not significant difference; * *p* < 0.05; ** *p* < 0.01; *** *p* < 0.001.

BMAL1 has been reported to have an important function in the regulation of apoptosis [29,30]. Consistently, our results showed that, compared with the C group, the proapoptotic mediator Bax and “apoptotic executor” Caspase3 expressions were increased in the periodontal tissue of the P group. In contrast, the antiapoptotic mediator Bcl-2 expression was decreased. Notably, the expression of apoptosis-related factors and the amounts of TUNEL-positive cells were increased in the periodontal tissues in the CRD-C group than in the C group, suggesting that CRD may cause increased apoptosis levels. Furthermore, the Bax and Caspase3 expression levels were upregulated, and the amounts of TUNEL-positive cells were raised in the periodontal tissues of the CRD-P group, suggesting that CRD may exacerbate periodontitis by increasing apoptosis levels through the regulation of BMAL1 (Figure 5A–C).

## 3. Discussion

CRD refers to the diurnal variation of the body’s physiological indicators, which is a physiological characteristic formed by the body’s adaptation to the rotation of the Earth [31]. However, CRD caused by shift work and sleep disturbances increases the danger of various systemic and metabolic diseases [32,33,34,35,36]. Recent studies have shown that biorhythmic clock disruption can destroy host defense responses and dysregulation of the immune system, which may be a pathogenesis mechanism that leads to various diseases [37,38]. BMAL1 is a core component of the circadian clock cycle, which directs the complex circadian expression of clock-controlled genes. In addition, it is directly modulated by the circadian clock system [39]. There is a tendency for loss of circadian biological clock function to disrupt the immune system, and downregulation of BMAL1 results in elevated expression of inflammatory mediators [40,41]. Active peripheral clock genes are present in periodontal tissues [42], while the role of clock genes in periodontal tissues remains poorly understood. Notably, periodontal tissues in the CRD group showed elevated levels of inflammation compared to group C, which we speculate may be related to the downregulation of BMAL1 signaling molecules due to the disruption of circadian homeostasis. It has been documented that BMAL1 knockout mice exhibit an inflammatory response [43]. It is generally accepted that the destruction of BMAL1 indirectly increases the NF-κB transcriptional activity and enhances the response to apoptosis [44,45]. In addition, our results showed that in the CRD-P group, alveolar bone resorption was more severe and the inflammatory response and osteoclast differentiation rose more significantly. Consequently, BMAL1 may be a key target to alleviate periodontitis by reducing the inflammatory response. 

The downregulation of BMAL1 signaling molecules is strongly correlated to various pathogenic factors, such as oxidative stress and inflammation. The imbalance between circadian rhythms and the ROS metabolic system may increase the damage caused by oxidative stress, which can lead to or complicate pathogenesis and aging [46]. Our data further suggest that the downregulation of BMAL1 molecular signaling can lead to the accumulation of ROS in periodontal tissues, as well as significant alterations in oxidative stress-related indicators. Therefore, we speculate that this may be related to the disruption of circadian homeostasis leading to the downregulation of BMAL1, which further elevates oxidative stress levels. It has been demonstrated that BMAL1 deficiency enhances LPS-induced activation of the NF-κB pathway by increasing ROS production [47]. These results suggest that CRD may exacerbate oxidative damage in periodontal tissues by downregulating the molecular expression level of BMAL1.

Meanwhile, BMAL1 also has an essential function in apoptosis. It has been demonstrated that BMAL1 suppresses the apoptosis level via modulating the Bax and Bcl-2 expression [29]. As is known, the upregulation of the proapoptotic mediator Bax and downregulation of the antiapoptotic mediator Bcl-2 and Caspase3 activation are inevitable in the onset of apoptosis. First, this study found that the expression of apoptosis-related factors was upregulated in the CRD-P group compared with the P group, suggesting that CRD can exacerbate the apoptotic response. We further observed that BMAL1 was downregulated in the CRD group compared with the C group, and the protein expression of apoptotic signaling markers was also upregulated, further demonstrating that BMAL1 downregulation exacerbated the apoptotic response. Furthermore, CRD may cause dysregulation of rhythmic gene expression, which further activates NF-κB signaling molecules and ultimately exacerbates periodontitis. In this sense, keeping the rhythm gene expression stabilized and restoring the normal circadian rhythm may be a feasible strategy for treating periodontitis. This study offers new insights into the complex pathophysiological mechanisms of periodontitis in terms of clock gene mechanism and provides novel strategies for disease prevention and treatment. However, in the present study, the role of BMAL1 in the progression of periodontitis was only preliminarily investigated by constructing a circadian rhythm disorder model, and our findings can be further validated in the future using a genetically defective animal model.

## 4. Materials and Methods

### 4.1. Animal

All animal experiments were conducted following the protocol approved by the Animal Ethics Committee of Jilin University (SY202207101). The minimum sample size was determined as eight motions per group using G*Power 3.1. software (power of 99%, bilateral 5% significance level, effect size of 0.9431542). Male Wistar rats (6 weeks old) were purchased from the Experimental Animal Center of Jilin University. During the domestication period, the animals were kept in stainless steel wire mesh cages and only healthy animals were assigned to the study. In the course of the experiment, All rats were housed under controlled temperature conditions at 22–25 °C, 55–70% humidity, and a 12-h light/dark cycle, and received unrestricted access to food and water.

During the experiment, 32 rats were randomly divided into four groups of eight rats, including control (C group), CRD without periodontitis (CRD-C group), periodontitis without CRD (P group), CRD with periodontitis (CRD-P group). In this study, we used a modified multiplatform approach to induce the CRD model [48,49]. As shown in Figure 1, The rats were placed in a tank with 14 circular platforms 6.5 cm in diameter, the narrow platforms were 12 cm apart from each other, so the rats could only stand on them, the tank was filled with water, the water surface was about 1 cm below the small platforms, the water temperature was kept at 18 ± 2°, and the water in the tank was changed daily. The four groups were treated in the same way, with the difference that the C group and P group placed metal nets on a small platform so that the rats could sleep on the nets. In the CRD-C group and CRD-P group, when the rats entered into rapid eye movement (REM) sleep, the loss of muscle tone caused the rats to come into contact with water, which led to the awakening of the rats. Thus, CRD was achieved by depriving the rats of paradoxical sleep. We referred to the Guidelines for the Care and Use of Mammals in Neuroscience and Behavioral Research laid down by the National Institutes of Health in the USA when Assigning scenarios to CRD models [50]. After the establishment of the circadian rhythm disorder model in rats, blood was taken from the right ventricle and serum CORT and ACTH levels were measured according to the ELISA kit instructions, and rats with significantly elevated CORT and ACTH (*p* < 0.05) were included in the circadian rhythm disorder group [25,26]. In addition, we conducted behavioral testing experiments. Then, a periodontitis model was constructed by ligating wires in the cervical region of the maxillary first molar in rats [51,52,53]. The rats were placed on a platform to acclimatize to their new environment before sleep deprivation. We placed rats on the platform for 18 h per day (14:00–8:00 +1 day). After the interruption of the circadian rhythm every 18 h, the animal can sleep for 6 h in a normal cage (starting at 8:00 a.m.). 

### 4.2. Enzyme-Linked Immunosorbent Assay (Elisa)

The concentrations of cortisol and ACTH in serum were determined by Elisa according to the kit instructions (Jianglai Biotechnology, Shanghai, China). 

### 4.3. Periodontal Clinical Index Examination

The gingival condition of rats in each group was observed under 2% pentobarbital sodium (0.2 mL/100 g) intraperitoneal anesthesia, and the gingival bleeding index (BI), probing depth (PD), and tooth mobility (TM) were examined and recorded. The methods and scoring criteria for clinical periodontal indicators were the same as we have described previously [41].

### 4.4. Micro-Computed Tomography (Micro-CT) Analysis

Scanning of the maxilla was performed with Micro-CT (μCT50, Scanco, Zurich, Switzerland). Micro-CT settings are as follows: 70 kV, 200 mA, and 300 ms (voxel size of 10 μm). For volumetric analysis, we selected 40 slices from the root part fork of the maxillary first molars. We assessed the relevant parameters, including bone mineral density (BMD), bone volume/total volume (BV/TV), and trabecular thickness (Tb.Th). The distance of the cement-enamel junction (CEJ) to the alveolar bone crest (ABC) was measured and averaged using Image J 8.0 (Image software, Bethesda, MD, USA).

### 4.5. Histopathological Staining

The maxilla was decalcified by placing it in a 10% ethylene diamine tetraacetic acid (EDTA) solution. After decalcification, the samples were routinely dehydrated and embedded in paraffin. Next, the samples were made into 5 μm paraffin sections along the long axis of the tooth. These paraffin sections will be subjected to hematoxylin-eosin staining and tartrate-resistant acidic phosphatase (TRAP) staining.

### 4.6. Detection of Oxidative Stress Levels

OS levels in periodontal tissues were estimated by detecting OS biomarker levels in serum. Elisa kits were used to determine the expression levels of superoxide dismutase (SOD) and malondialdehyde (MDA) (Jiancheng Institute of Biological Engineering, Shanghai, China).

### 4.7. Terminal Deoxynucleotidyl Transferase dUTP Nick End Labeling (TUNEL) Staining

Tissue biopsy specimens were collected and fixed from the connecting epithelium, sulcular epithelium, and part of the connective tissue on the crescent. Frozen sections (6 μm thick) were taken for apoptosis detection. Apoptotic cells were detected in periodontal tissues using the TUNEL kit (Beyotime, Shanghai, China).

### 4.8. Mito SOX Staining

Mito SOX selectively reacts with superoxide in mitochondria and is used to measure ROS production in mitochondria. Fresh gingiva around the maxillary first molars was collected and frozen sections were made. The frozen sections were then incubated with Mito SOX Red reagent (Thermo Fisher Science, Waltham, MA, USA). Afterward, the nuclei were stained with a Hoechst staining solution (Sigma, St. Louis, MO, USA). Finally, the results were observed under a fluorescent microscope. 

### 4.9. Immunohistochemical Analysis

Immunohistochemistry was performed to detect the protein expression of BMAL1, NF-κB, 8-OHdG, and SOD1 in the tissues. After dewaxing and gradient dehydration, antigen repair was performed with gastrin repair solution followed by blocking with normal goat serum. Primary antibodies against BMAL1 (1:300, Abcam, Cambridgeshire, Cambs, UK), NF-κB (1:200, Cell Signaling Technology, Danvers, MA, USA), 8-OHdG (1:100, Abcam, Cambridgeshire, Cambs, UK), and SOD1 (1:100. Abcam, Cambridgeshire, Cambs, UK) primary antibodies were incubated overnight at 4 °C. Sections were washed with phosphate-buffered saline and then incubated with secondary antibodies (ZSGB-BIO, Beijing, China). Sections were incubated with horseradish peroxidase-labeled streptavidin and 3,3-diaminobenzidine. Finally, re-staining was performed with hematoxylin.

### 4.10. qRT-PCR

The experimental procedure of qRT-PCR was as previously reported [53]. The relative gene expression was calculated by the 2^−ΔΔCt^ method. The primer sequences involved are listed in Table 1.

### 4.11. Western Blot Analysis

Western blot methods and procedures were as previously reported [52]. Primary antibody information is as follows: BMAL1 (1:1000, Abcam, Cambridgeshire, Cambs, UK), NF-κB (1:2000, Cell Signaling Technology, Danvers, MA, USA), Casepase3 (1:2000, Cell Signaling Technology, Danvers, MA, USA), Bax (1:2000, Proteintech, Rosemont, IL, USA), Bcl-2 (1:2000, Proteintech, Rosemont, IL, USA) and β-actin (1:8000, Proteintech, Rosemont, IL, USA). The grayscale values were analyzed by using Image J 8.0 (Image software, Bethesda, MD, USA).

### 4.12. Statistical Analysis

All data are reported as mean ± standard deviation. The Shapiro–Wilk test was used to assess the normality of data. In the case of data with normal distribution, An unpaired Student’s *t*-test analyzed the differences between the two groups, and the differences between multiple groups were analyzed using one-way ANOVA. Statistical analysis using GraphPad Prism 8.0. Statistical significance was set at *p* < 0.05.

## 5. Conclusions

Collectively, the study indicates that BMAL1 may have a crucial role in periodontitis development. CRD downregulates BMAL1 expression, thereby indirectly increasing NF-κB transcriptional activity, resulting in a remarkable increase in oxidative stress and apoptosis, which ultimately exacerbates periodontal tissue destruction. Therefore, clock-related BMAL1 may be an important target in treating periodontitis. Although BMAL1 plays a central role in the regulation of the circadian biological clock, other biological clock proteins, such as CLOCK, CRY and PER genes, also play important roles in regulating circadian homeostasis. Moreover, their potential mechanisms in the progression of periodontitis deserve further investigation.

## Figures and Tables

**Table 1:** Primer sequences for qPCR.

Gene	Gene Accession No.	Primer Sequence (5′–3′)
BMAL1	NM_024362.2	F: GCTTTGAGGTGACCAGCAAGTACA
		R: AAGGGCTCCAAGGTCCACAG
NF-κB	NM_199267.2	F: CGACGTATTGCTGTGCCTTC
		R: TTGAGATCTGCCCAGGTGGTA
Bax	NM_017059.2	F: CCACCAAGAAGCTGAGCGA
		R: GCTGCCACACGGAAGAAGA
Bcl-2	NM_016993.2	F: TTGAGTTCGGTGGGGTCATG
		R: GATCCAGGTGTGCAGATGCC
Caspase3	NM_012922.2	F: AGCCGAAACTCTTCATCATTCA
		R: CCATATCATCGTCAGTTCCACT
β-actin	NM_031144.3	F: GGAGATTACTGCCCTGGCTCCTA
		R: GACTCATCGTACTCCTGCTTGCTG

## Data Availability

Data available on request due to restrictions, e.g., privacy or ethical. The data presented in this study are available on request from the corresponding author.
